# Posttranslational modifications and secretion efficiency of immunogenic hepatitis B virus L protein deletion variants

**DOI:** 10.1186/1743-422X-10-63

**Published:** 2013-02-25

**Authors:** Baiba Niedre-Otomere, Ance Bogdanova, Ruta Bruvere, Velta Ose, Wolfram H Gerlich, Paul Pumpens, Dieter Glebe, Tatjana Kozlovska

**Affiliations:** 1 Biomedical Research and Study Centre, Ratsupites street 1, LV-1067, Riga, Latvia; 2 Institute of Medical Virology, Justus Liebig University, Schubertstr. 81, D-35392, Giessen, Germany

**Keywords:** L protein, PreS1 domain, N-terminal myristoylation, Secretion, Glycosylation

## Abstract

**Background:**

Subviral particles of hepatitis B virus (HBV) composed of L protein deletion variants with the 48 N-terminal amino acids of preS joined to the N-terminus of S protein (1-48preS/S) induced broadly neutralizing antibodies after immunization of mice with a Semliki Forest virus vector. A practical limitation for use as vaccine is the suboptimal secretion of such particles. The role of the N-terminal preS myristoylation in the cellular retention of full-length L protein is described controversially in the literature and the relation of these data to the truncated L protein was unknown. Thus, we studied the effect of preS myristoylation signal suppression on 1-48preS/S secretion efficiency, glycosylation and subcellular distribution.

**Findings:**

The findings are that 1-48preS/S is secreted, and that removal of the N-terminal myristoylation signal in its G2A variant reduced secretion slightly, but significantly. The glycosylation pattern of 1-48preS/S was not affected by the removal of the myristoylation signal (G2A mutant) but was different than natural L protein, whereby N4 of the preS and N3 of the S domain were ectopically glycosylated. This suggested cotranslational translocation of 1-48preS in contrast to natural L protein. The 1-48preS/S bearing a myristoylation signal was localized in a compact, perinuclear pattern with strong colocalization of preS and S epitopes, while the non-myristoylated mutants demonstrated a dispersed, granular cytoplasmic distribution with weaker colocalization.

**Conclusions:**

The large deletion in 1-48preS/S in presence of the myristoylation site facilitated formation and secretion of protein particles with neutralizing preS1 epitopes at their surface and could be a useful feature for future hepatitis B vaccines.

## Introduction

Hepatitis B virus (HBV) contains three closely related transmembrane proteins in its lipid envelope. The S protein comprises the common C terminus of all three HBV surface (HBs) proteins and is the main structural component of the viral envelope. The nonessential M protein contains a preS2 N-terminal extension of 55 amino acids (aa), while the essential L protein has a further N terminal extension of 108 or 119 aa, termed preS1 [1]. The preS1 domain contains at its N-terminus an essential attachment site of HBV to infection-susceptible cells [2] while the preS2 sequence seems to have no essential function in the viral life cycle beyond acting as a spacer in the L protein [3]. HBs proteins are secreted from infected, transfected, or transduced cells as 20–22 nm spherical or filamentous, noninfectious lipoprotein particles.

During translation, the preS domain (i. e. preS1 + preS2) of L protein remains unglycosylated and in the cytosol whereas the S domain is partially N-glycosylated and assumes a topology at the endoplasmatic reticulum (ER) with at least two transmembrane passes [4]. However, in a posttranslational maturation step approximately 50% of the preS domains are translocated to the lumen of secretory structures and appear later at the surface of the secreted HBV or subviral particles while the cytosolic domains remain within the particles [5-7]. Full length L protein alone in absence of the S protein is not secreted from transfected cells [8-10]. The retention of L protein depends on the cytosolic localization of preS1 [11], but N-terminal shortening of the preS sequence by more than 110 aa (or 98 aa in HBV genotype D) finally leads to its cotranslational translocation to the ER [11] governed by the signal peptide I [4] of the S domain, N-glycosylation of the residual preS sequence [12] and secretion of subviral particles.

The 48 aa N-terminal part of preS1 carries HBV-neutralizing epitopes [2,13] while the C-terminal part does not [14]. Furthermore the C-terminal part in its cytosolic orientation prevents secretion [15]. These observations led to the design of expression vectors encoding the N-terminal part of preS1 linked with the S protein for the generation of potential HBV vaccines [16,17]. Using replication-deficient Semliki Forest virus (rSFV) vectors, we could express the neutralization-relevant preS1 part linked to the S domain of HBs subviral particles and consequently use these vectors for immunization of mice [18]. The influence of the large internal deletion in the L protein on its structure and topology was not yet known. Therefore, we analyzed the cellular localization, the N-glycosylation pattern of expressed 1-48preS/S protein variants, and the surface exposure of the shortened preS1 domain on the subviral particles.

Furthermore, secretion of the subviral particles from the transduced cells was found to be a limiting factor in our previous study. Therefore, we searched for ways to improve the secretion of the HBs particles. The N–terminus of the L protein is modified by myristate at Gly 2 of preS1 [19]. Kuroki et al. [20] found that myristoylation alone did not cause retention, but Prange et al. reported the opposite [21]. More recently, Abou-Jaoude et al. [22] studied the secretion and infectivity of hepatitis delta virus variants with and without N-terminal myristoylation of L and other HBV envelope proteins. They found no detectable influence of the myristoylation on the secretion of these viruses but could show that infectivity was lost after inactivation of the myristoylation signal in L protein. It was not clear how these findings could be applied to the context of the 1-48preS/S particles with a heavily truncated preS domain. Thus, it appeared useful to determine whether inactivation of the myristoylation signal would improve secretion of the 1-48preS/S protein and allow for surface exposure of the preS1 epitopes. Exchange of Gly2 to Ala (G2A) disrupts the myristoylation motif Met-Gly recognized by N-myristoyl transferase [23] and this mutation was introduced in an expression vector for generation of L protein deletion variants without a myristoylation signal.

## Results and discussion

**Figure 1:**
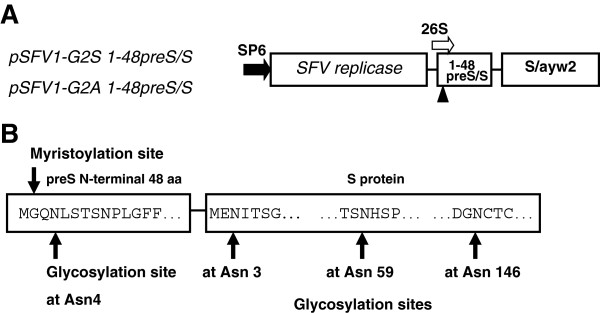
**A. Schematic representation of the SFV expression vectors.** SP6 RNA polymerase promoter for transcription *in vitro* is shown by the filled arrow. Sequences encoding 1-48preS/S variants are placed under the control of SFV 26S subgenomic promoter (empty arrow) and expression is directed by SFV replicase. The filled triangle denotes the modified myristic acid attachment site, where Gly2 was replaced with Ala or Ser in L deletion variants G2A 1–48preS/S and G2S 1-48preS/S. The space between the regions encoding aa 1–48 of preS1 and S/ayw2 denotes the “spacer” encoding aa LEGGSGG. **B**. Schematic representation of L protein deletion variants consisting of the first 48 aa of preS1 fused to the N-terminus of the S domain showing the myristoylation and potential glycosylation sites.

Generation of rSFV vector plasmids (pSFV1) encoding the 1-48preS/S variants with the myristic acid attachment site have been described [18]. Its variant with Gly 2 substituted to Ser (pSFV1-G2S 1-48preS/S) (Figure 1A) was generated by amplification of the corresponding gene from the plasmid pFD Pr[13–59]S (kindly provided by K. Sasnauskas) by forward primer 5^′^ GACACAGATCTGCCGCCACCATGTCTCAGAATCTTTCCAC 3^′^; the G2A variant (pSFV1-G2A 1-48preS/S) (Figure 1A) was generated by forward primer 5^′^ GACACAGATCTGCCGCCACCATGGCCCAGAATCTTTCCAC 3^′^ from plasmid pFD GlyPr[13–59] (gift from K. Sasnauskas). The reverse primer for generation of both plasmids was 5^′^ CTCTGTACCCGGGTTATTAGTGATGGTGATGGTGATGAATG 3^′^. The fragments were cloned in the pSFV1 vector at the BglII and SmaI sites and insertions confirmed by sequencing. Replication-deficient rSFV [24] were produced by co-electroporation of BHK-21 cells with an *in vitro* transcribed vector and helper RNA. Huh7 cells were infected with rSFV at MOI 10, cell medium was replaced after 18 h with a fresh medium, which was collected after 24 h and cells were lysed with 0.5% Triton X-100 lysis buffer. Cell medium and lysates were subjected to in-house ELISAs as described [18] with monoclonal antibodies (MAbs) MA18/7 recognizing epitope DPAF of preS1 20–23 in genotype D and C20/02 recognizing the correctly folded S domain between aa 118 and 149 (W. H. Gerlich, unpublished).

The secretion of the 1-48preS/S protein variants is shown in Table 1. We observed a slightly but significantly reduced secretion of the 1-48preS/S variant with an inactivated myristoylation site (G2A or G2S) compared to the unmodified variant, although the intracellular expression level of the wt and the G2S mutant was equal (Table 1). The difference is small but the accuracy of the immune assays used suggests that the inactivation of the myristoylation signal had indeed a minor negative effect on the release of the particles. The data are compatible with the report of Abou-Jaoude et al. [22] who did not observe a difference of HDV secretion with or without myristoylation as detected by qualitative immunoblot.

**Table 1:** Secretion of L protein deletion variants

	**Secreted, OD**_**492**_		**Intracellular, OD at 492 nm**	Ratio secreted/intracellular*
**L deletion variant**	**MAbs**			
	**MA18/7**	**C20/02**	**MA18/7**	
1-48preS/S, myr wt	1.10 ± 0.16	1.00 ± 0.12	1.08 ± 0.08	1.06 ± 0.09
G2A 1-48preS/S, myr^-^	0.52 ± 0.06	0.56 ± 0.09	0.69 ± 0.04	0.78 ± 0.04**
G2S 1-48preS/S, myr^-^	0.67 ± 0.08	0.43 ± 0.06	1.14 ± 0.09	0.60 ± 0.06**
1-48preS/S_0_, myr wt	0.17 ± 0.01	0.14 ± 0.01	0.72 ± 0.10	0.25 ± 0.07

Huh7 cells were infected at MOI 10 with rSFV encoding respective proteins. 18 h after infection cell medium was collected and replaced with a fresh medium. This was collected after 24 h and cells were lysed with a buffer containing 0.5% Triton X-100, 150 mM NaCl, 50 mM Tris–HCl pH 7.5, 2 mM EDTA and 1 μg/ml phenylmethanesulfonylfluoride. Huh7 cell lysates with 5 μg/ml cell protein and 100 μl of 2 ml of medium were analyzed by ELISA on microtiter plates coated with MAbs MA18/7 and C20/02. OD_492_ of cell lysates tested on mictrotiter plate coated with C20/02 was barely above cut-off (OD at 492 nm = 0.1) for all 1-48preS/S proteins and therefore not shown. Mean values are shown ± SD.

* Ratios were calculated with OD_492_ values obtained with ELISA using MAb MA18/7.

** The difference to 1-48preS/S myr wt is significant at α = 0.05.

**Figure 2:**
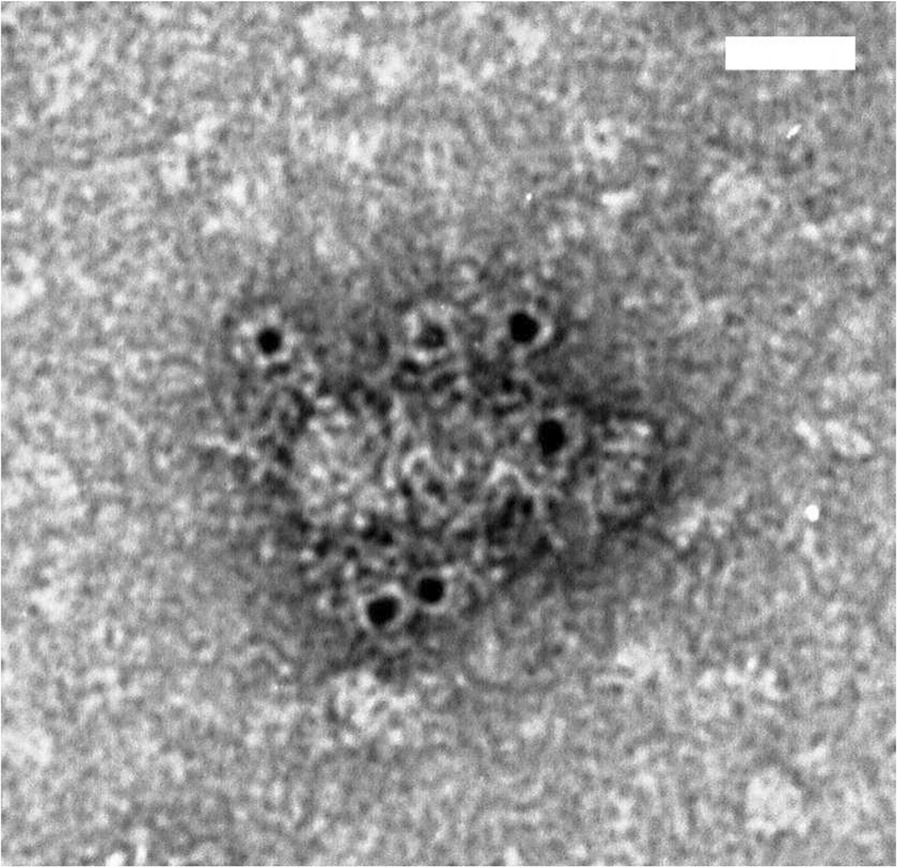
**Electron microscopy analysis of immunogold-labelled G2S mutant of 1-48preS/S subviral particles after reaction with MAb MA18/7 recognizing preS1.** The suspension of the particles was adsorbed on carbon-formvar coated grids and incubated with MAb MA 18/7, followed by anti-mouse IgG conjugated with 5 nm gold particles (Sigma), as described [24]. The grids were negatively stained with 2% uranyl acetate aqueous solution and examined with a JEM-1230 electron microscope (JEOL Ltd.,Tokyo, Japan) at 100 kV. The scale bar corresponds to 25 nm.

By electron microscopy of concentrated Huh7 cell medium 22 h after infection we could confirm that the G2S variant was released as 22 nm subviral particles with an accessible preS1 antigen on the surface as shown by binding of MAb MA18/7 and subsequent anti-mouse IgG conjugated with 5 nm gold particles (Figure 2) according to the method of Louro and Lesemann [25]. To rule out that these particles were released by cell lysis due to transduction with the apoptosis-inducing rSFV vector, Huh7 cells were transduced with rSFV encoding full length L protein expressing also S and M (not shown) and the secretion incompetent variant 1-48preS/S_0_ which lacks the start codon of S protein [18]. In case of the 1-48preS/S_0_ variant, the MA18/7-specific signal from the cell medium was barely above the cut-off (Table 1), while no MA18/7-specific signal was found in the cell medium of L protein transduced cells (not shown).

**Figure 3:**
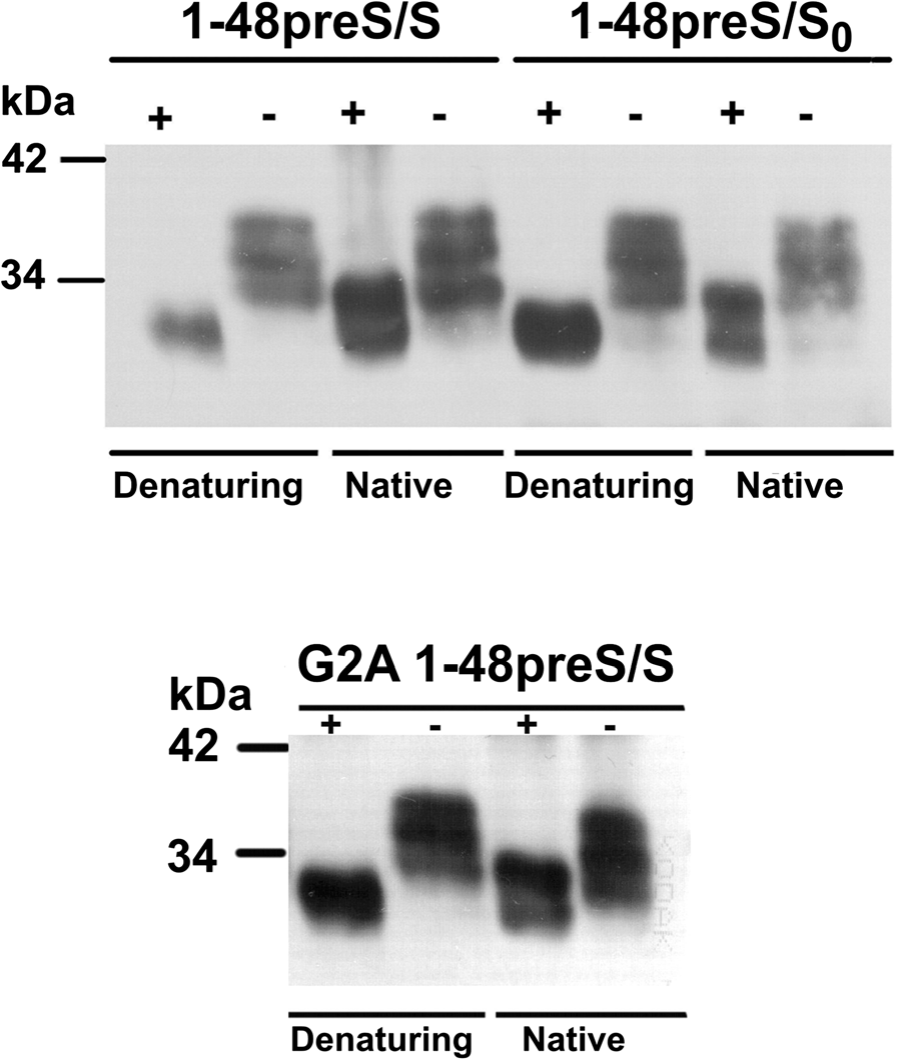
**N-Glycosylation pattern of L protein deletion variants.** Huh7 cells were infected at MOI 10 with rSFV encoding 1-48preS/S, G2A 1-48preS/S and 1-48preS/S_0_. Twenty hours after infection cells were lysed with lysis buffer containing 0.5% Triton X-100, 150 mM NaCl, 50 mM Tris–HCl pH 7.5, 2 mM EDTA and 1 μg/ml phenylmethanesulfonylfluoride. Five-hundred U of PNGase F were added to 10 μl of Huh7 cell lysates diluted in reaction buffer (50 mM sodium phosphate buffer, pH 7.5) containing 1% NP40, and incubated for 1 h at 37°C. To perform the reaction under denaturing conditions, glycoprotein denaturing buffer was added to 10 μl of cell lysates and incubated for 10 min at 100°C, whereas for digestion under native conditions denaturing buffer was omitted. After separation by SDS-PAGE, proteins were transferred to a Hybond-P membrane in a semi-dry electro blotter. The membrane was reacted with MAb MA18/7, followed by goat anti-mouse antibodies conjugated with horseradish peroxidase. “+”, treated with PNGase F; “-”, untreated.

Western blot analysis with MA18/7 of Huh7 cell lysates revealed three L protein-related bands at approximately 32, 35 and 38 kDa. PNGase F digestion under denaturing conditions shifted the three bands to one 30 kDa position (Figure 3). This suggested that the 1-48preS/S variants existed as triple, double and single N-glycosylated forms, in contrast to wt L protein which exists in a major single glycosylated and a minor unglycosylated form. The 1-48preS/S variants bear four potential N-glycosylation sites: N4 in the preS1 fragment, and N3, N59 and N146 in the S domain (Figure 1B). N146 is partially used in all 3 HBs proteins [1], while N3 of the S domain or S protein is not glycosylated in natural HBs proteins. According to the transmembrane topology [26], we assume that the 1-48preS/S variants are partially glycosylated at N3 of the S domain, whereas N59 is most probably within the cytosolic loop and not accessible. In full-length L protein, preS is not translocated to the ER and should be unglycosylated [5], because preS1 lacks an N-terminal signal peptide for cotranslational translocation [6,11]. In line with this model, only O-glycosylation of preS2 but no preS-N-glycosylation was found in natural L-containing HBsAg from HBV carriers [27]. A posttranslational N-glycosylation of N 4 and N112 (genotype D) in preS1was reported more recently in full-length L protein expressed in various transfected cell lines [26]. In contrast, the postulated glycosylation at N4 of preS1 in the L deletion variants studied here is in line with data obtained with L protein devoid of aa 70 – 107 of preS1 [16]. In order to discriminate between preS1 and S linked glycans, PNGase F digestion was also performed in the absence of glycoprotein denaturing buffer in order to preserve the native conformation of S protein. N146-linked glycan is protected from digestion in the native conformation of the S domain whereas preS glycans are still removed [28]. Under these conditions, the major product had 32 kDa, but the nonglycosylated 30 kDa product was also observed. This suggests that the majority of proteins is glycosylated at N146 and at one or both of the other sites. Furthermore, it shows that at least one site upstream of signal peptide I i. e. N3 in S or N4 in preS1 may be occupied even in absence of glycan at N146 in S. The glycosylation pattern of the in-frame co-expressed S protein was not affected, S protein could be detected in single glycosylated and unglycosylated form for the G2A and G2S 1-48preS/S mutants. Glycosylation of the non-secreted 1-48preS/S_0_ protein did not differ from the 1-48preS/S variant. Likewise, Prange et al. reported secretion and glycosylation at N4 of a protein consisting of the first 41 aa of preS1 (subtype ayw) joined N-terminally to the S protein [16].

The influence of myristoylation of L protein on secretion has been found to be different in various studies [20,22] suggesting a potential ER retention [21]. Thus, we studied the secretion of the 1-48preS/S_0_ variant by inactivation of the myristoylation site but particles were again not secreted (not shown). The N-glycosylation pattern of the S_0_ variant was unaltered and was obviously not related to secretion competence. Theoretically, the postulated glycosylation at N3 of the S domain could enhance secretion as shown for glycosylation of M protein [29], but this was not the case for the 1-48preS/S_0_ variant. Based on previous data [22] we conclude that the major determinant of effective secretion of wt and modified L protein subviral particles is the in-frame co-expressed S protein.

Surprisingly, presence of the myristoylation signal did not impair the secretion of 1-48preS/S particles but improved it slightly (Table 1). The somewhat better secretion of these particles with the myristoylation signal is also in agreement with reports that myristoylation supports virus-like particle formation of Lassa virus [30] and virus assembly of HIV [31]. Although, myristoylation is reported to be dispensable for virion morphogenesis of HBV [32], its role and mechanisms in facilitating generation of subviral particles may be a subject of future investigation.

**Figure 4:**
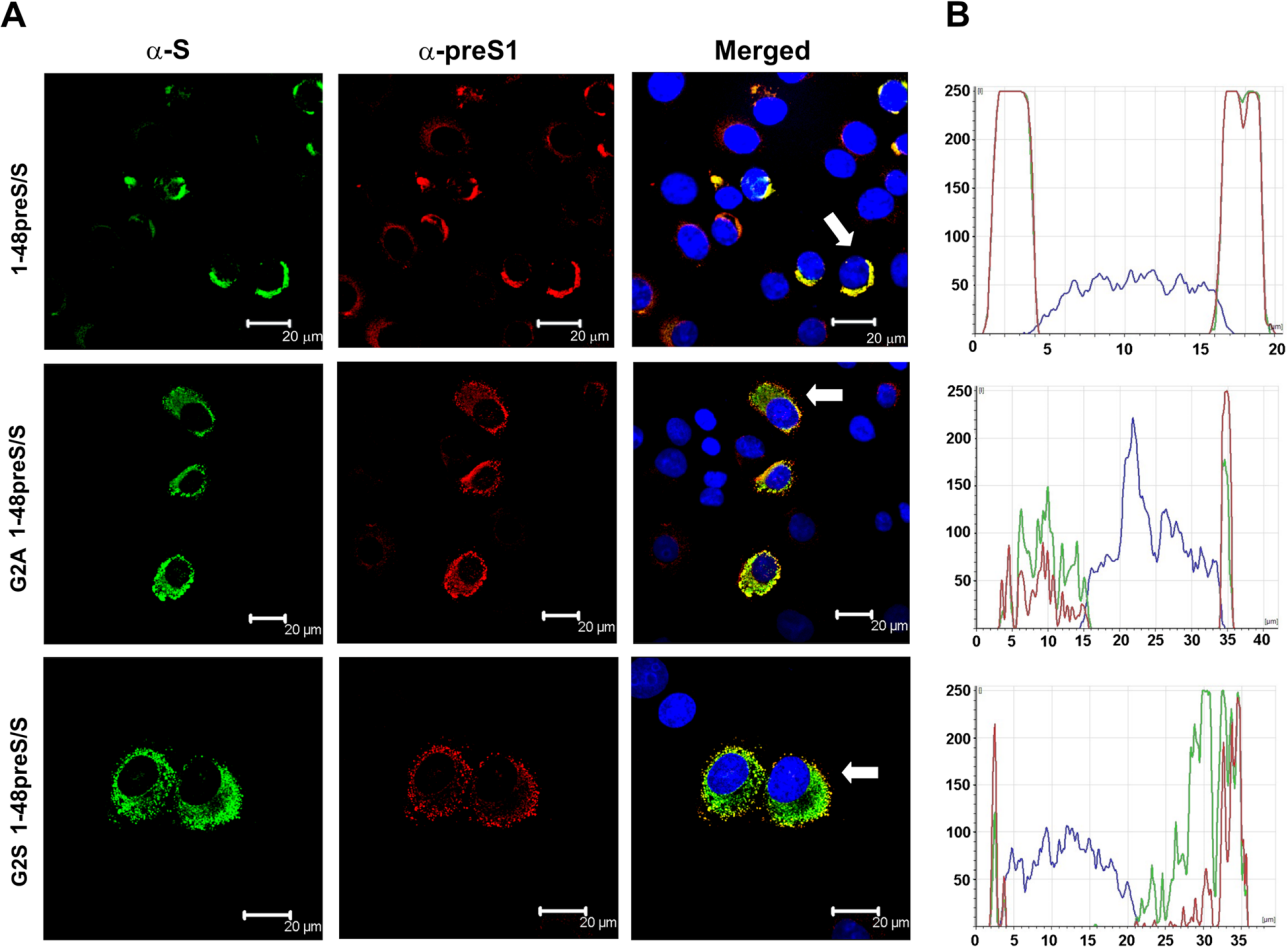
**Confocal microscopy analysis of 1-48preS/S, G2A 1-48preS/S and G2S 1-48preS/S proteins.** BHK-21 cells were infected with rSFV encoding the respective proteins as described in Materials and Methods section and stained with MAb 1-9C1 (α-S), followed by polyvalent rabbit antibodies H863 (α-preS1). The cell nuclei were stained with DAPI. Panel **A** depicts images of cells, panel **B** shows the fluorescence intensity of each fluorophore - FITC, TRITC and DAPI (y axis, relative numbers) along a line (x axis, μm) drawn across a selected cell which is marked by the white arrow.

Myristoylation serves as a membrane anchor for a number of proteins [33]. To examine how mutation of the myristoylation site affects the subcellular localization of L protein deletion variants, BHK-21 cells were infected with rSFV encoding 1-48preS1/S, G2S and G2A mutants and were analyzed by confocal immunofluorescence microscopy. Cells were stained with S specific MAb 1-9C1 directed to a linear epitope in the S domain followed by preS1 specific polyclonal antibodies H863. The anti-S antibody binds to the S domain in L protein deletion variants as well as to in-frame co-expressed S protein. In rSFV-1-48preS/S transduced cells anti-S and anti-preS1 signals were mostly localized in the perinuclear area with a strong colocalization of both antigens (Figure 4, upper panel of sections A and B). In contrast, the Gly2 mutants showed a dispersed localization throughout the cell cytoplasm and a weak colocalization of the two antigens (Figure 4 middle and bottom panels of sections A and B). These results are in line with the matrix protein Z of Lassa virus where mutation of N-terminal Gly to Ala altered the subcellular localization from a punctuate to a diffuse pattern [30]. The stronger colocalization of the preS1 and S epitopes in cells transfected with the wt variant than with the variant without myristoylation signal suggests that the myristate supports the interaction between the L protein and the S protein and this would probably increase secretion.

## Competing interests

Authors declare that they have no competing interests.

## Authors’ contributions

BNO produced rSFV vectors, performed cell infection with these vectors, participated in the design of the study, collated and analyzed the data and wrote the manuscript. AB performed cell infection with rSFV, immunoassays, PNGase F digestion and Western blotting. RB performed confocal microscopy analysis. VO provided electron microscopy images. PP revised the manuscript and gave final approval of the version to be published. DG and WHG participated in the design of the study, provided MAbs, assisted in the interpretation of the results, the writing and revision of the manuscript. TK participated in the design of the study and generation of pSFV1 plasmids encoding L protein deletion variants. All of the authors read and approved the final version of the manuscript.
